# Radial Peripapillary Capillary Density Involved in Nasal Optic Disc Thinning and Visual Field Abnormalities Using Optical Coherence Tomography Angiography

**DOI:** 10.3390/tomography12050073

**Published:** 2026-05-15

**Authors:** Miki Yoshimura, Yuki Hashimoto, Yuko Kodama, Aris Hatanaka, Ryusei Yakushiji, Shiho Ikeda, Nazuna Inoue, Maho Wakabayashi, Ichika Kawazu, Takeshi Yoshitomi

**Affiliations:** 1 Department of Orthoptics and Visual Sciences, Faculty of Health and Medical Sciences, Graduate School of Health and Welfare Sciences, International University of Health and Welfare, Sawara-ku, Fukuoka 814-0001, Japan; yoshimura.m08@gmail.com (M.Y.); yoshitomi@ihwg.jp (T.Y.); 2 Department of Orthoptics, Faculty of Medicine, Fukuoka International University of Health and Welfare, Sawara-ku, Fukuoka 814-0001, Japan; 2213017@s.takagigakuen.ac.jp (Y.K.); 2213030@s.takagigakuen.ac.jp (A.H.); 2213040@s.takagigakuen.ac.jp (R.Y.); 2113002@s.takagigakuen.ac.jp (S.I.); 2113006@s.takagigakuen.ac.jp (N.I.); 2213044@s.takagigakuen.ac.jp (M.W.); 2413008@s.takagigakuen.ac.jp (I.K.)

**Keywords:** circumpapillary retinal nerve fiber layer thickness, laser speckle flowgraphy, mean blur rate, optical coherence tomography angiography, radial peripapillary capillary

## Abstract

Objectives: This study investigated whether visual field abnormalities are present in eyes with suspected nasal optic disc hypoplasia (NOH) by using fundus photography and optical coherence tomography (OCT). Methods: NOH was diagnosed using the following criteria: (1) small optic disc, (2) nasal optic disc pallor or optic disc margin irregularity, (3) wedge-shaped temporal visual field defects extending from Mariotte’s blind spot, and (4) reduced nasal circumpapillary retinal nerve fiber layer (cpRNFL) thickness. Eyes fulfilling criteria 1, 2, and 4 without visual field abnormalities were classified as pseudo-NOH (pNOH), whereas eyes without visual field or cpRNFL abnormalities were considered normal. Nasal cpRNFL thickness was measured using OCT, radial peripapillary capillary (RPC) density was assessed using OCT angiography (OCTA), visual field testing was performed, and optic disc blood flow velocity was evaluated using the mean blur rate (MBR) and laser speckle flowgraphy (LSFG). Results: Seven eyes with NOH, 13 eyes with pNOH, and 24 normal right eyes were included. Nasal cpRNFL thickness and MBR were significantly reduced in both the NOH and pNOH groups compared with the normal group, with no significant difference between the NOH and pNOH groups. Nasal RPC density was significantly lower in the NOH group than in both the pNOH and normal groups, and no significant difference was observed between the pNOH and normal groups. Conclusions: Even when NOH was suspected from fundus, LSFG, and OCT C-scan findings, visual field abnormalities were not consistently present. Differences in RPC density measured using OCTA may have contributed to this variability. This study examined whether suspected nasal optic disc hypoplasia (NOH) is always associated with visual field defects. Using fundus imaging, OCT, OCT angiography, and laser speckle flowgraphy, we compared eyes with NOH, pseudo-NOH, and normal eyes. Although structural changes such as reduced nasal nerve fiber layer thickness and decreased blood flow were observed in both NOH and pseudo-NOH, visual field abnormalities were not consistently present. Notably, reduced radial peripapillary capillary density was specific to NOH, suggesting that vascular differences may explain variability in visual function. These findings highlight the importance of multimodal imaging in NOH evaluation.

## Simple Summary

This study examined whether suspected nasal optic disc hypoplasia (NOH) is always associated with visual field defects. Using fundus imaging, optical coherence tomography (OCT), OCT angiography, and laser speckle flowgraphy, we compared eyes with NOH, pseudo-NOH, and normal eyes. Although structural changes such as reduced nasal nerve fiber layer thickness and decreased blood flow were observed in both NOH and pseudo-NOH, visual field abnormalities were not consistently present. Notably, reduced radial peripapillary capillary density was specific to NOH, suggesting vascular differences may explain variability in visual function. These findings highlight the importance of multimodal imaging in NOH evaluation.

## 1. Introduction

Optical coherence tomography (OCT) and OCT angiography (OCTA) are useful for analyzing and evaluating the optic disc for diagnosis, follow-up, and understanding the pathology of glaucoma and optic disc hypoplasia [1,2,3,4].

Nasal optic disc hypoplasia (NOH), a type of optic nerve hypoplasia, is known to cause abnormalities in the morphology and circulatory dynamics of the nasal optic nerve papilla and its peripapillary area [3,4,5,6,7]. Circumpapillary retinal nerve fiber layer (cpRNFL) thickness is measured using OCT C-scans, and the thinning of the nasal cpRNFL can be clearly observed in the area consistent with a visual field abnormality on the temporal side of the NOH [3,4,5,6]. Glaucomatous eyes with temporal visual field abnormalities similar to those of NOH eyes have a slightly thinner nasal cpRNFL than NOH eyes, which is helpful in differentiating between the two conditions [5].

A previous report measuring optic nerve head blood flow by using laser speckle flowgraphy (LSFG) found that the blood flow velocity in the nasal side of the optic disc in NOH eyes was reduced, consistent with the nasal region where the cpRNFL was thinner and had visual field abnormalities [3]. The NOH also showed a significant positive correlation with nasal cpRNFL thickness and nasal optic disc blood flow velocity [3]. Therefore, the assessment of cpRNFL thickness by OCT and blood flow velocity in the optic disc with LSFG is useful for NOH [3,4,5,6].

Recently, a report was published on the use of OCTA to show a decrease in nasal papillary radial peripapillary capillary (RPC) density in cases of NOH [4]. However, the relationships between decreased nasal cpRNFL thickness, nasal optic disc blood flow velocity, nasal RPC density, and visual field abnormalities have not yet been clarified. Furthermore, during ophthalmological examination, NOH may be suspected on the basis of fundus photography and C-scan images obtained using OCT. However, no study has examined the presence or absence of visual field abnormalities on the basis of RPC density and blood flow velocity. Therefore, this study aimed to investigate in detail the relationship among the optic disc, its surrounding blood flow circulation and morphology, and the visual field in eyes with suspected NOH.

## 2. Materials and Methods

### 2.1. Study Design and Participants

This prospective cross-sectional study included healthy Japanese adults who were recruited via a nonprobability sampling method by using posted notices. All participants had a best-corrected visual acuity (BCVA) of 20/20 or better, no history of cardiovascular or ophthalmic disease, and had not received any ophthalmic or systemic medical treatment. Post hoc tests showed that our sample size was sufficient, with a statistical power of 99.9% (1 − β = 0.99).

### 2.2. Ethics Approval

The study adhered to the principles of the Declaration of Helsinki. The research protocol was approved by the Ethics Committee of Fukuoka International University of Health and Welfare on 19 January 2024 (Approval No. 23-fiuhw-012). All participants provided written informed consent.

### 2.3. Ocular Examinations

All participants underwent refractive examination; BCVA, intraocular pressure (IOP), and axial length (AL) assessments; fundus photography of the posterior pole; and cpRNFL thickness measurement on the C-scan of spectral domain OCT (RS-3000 Advance 2; Nidek Co., Ltd., Gamagori, Japan) of the optic disc. A Humphrey Field Analyzer static visual field test (24-2 Swedish Interactive Threshold Algorithm Standard) was also performed. The ratio of the disc–macula distance to the disc diameter (DM/DD) was determined for the size of the optic disc by using fundus photographs. The DM/DD measurements were performed as previously described. The fovea centralis was marked. The horizontal distance (*b*) between the fovea centralis and the temporal margin of the optic disc was measured, as well as the horizontal (*a*l) and vertical (*a*2) optic disc diameters. When the “double ring sign” was present, the margins of the “inner ring” provided the reference points. The DM/DD ratio was estimated for each case by using the following formula: DM/DD = (half the horizontal disc diameter + the distance between the fovea and disc margin) + the mean disc diameter (1) [8].


(1) $$\frac{\frac{a1}{2}+b}{\frac{a1+a2}{2}}.$$

**Figure 1:**
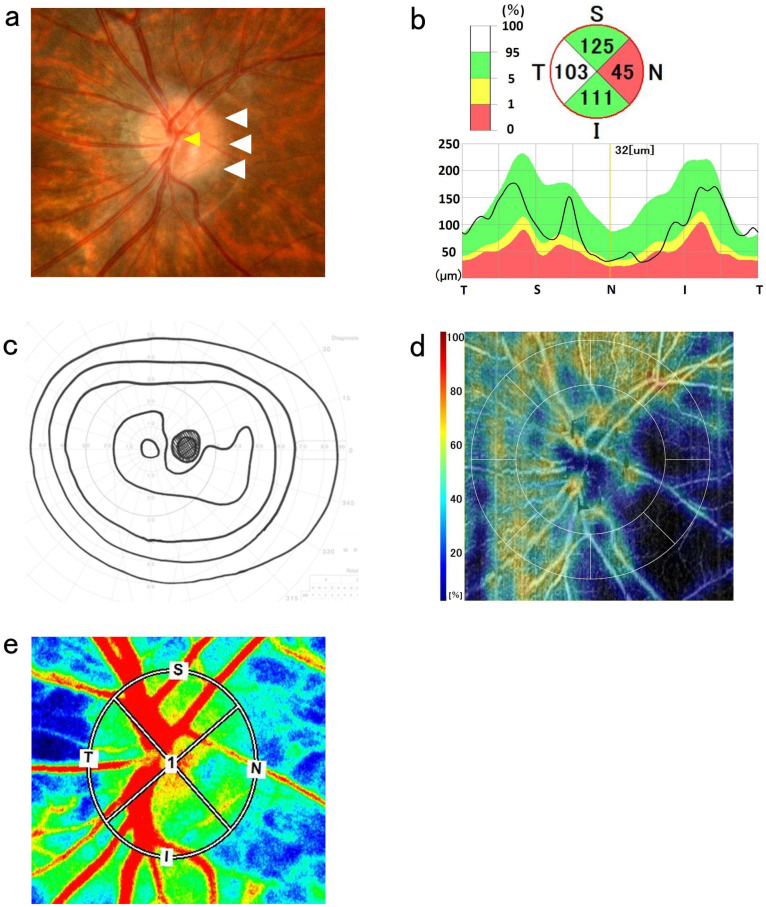
Images showing nasal optic disc hypoplasia (NOH) in the right eye. (**a**) Fundus photography reveals a double ring-shaped appearance of the nasal optic disc (white arrowheads) and a small optic disc. It also shows inverted disc vessels (yellow arrowhead). (**b**) The thickness of the circumpapillary retinal nerve fiber layer using optical coherence tomography is thinner in the nasal region, as indicated by the abnormal values shown in red color. (**c**) A wedge-shaped temporal visual field defect was observed using the Goldman perimetry test. (**d**) The color map of radial peripapillary capillary (RPC) density shows markedly reduced RPC density in the nasal region of the NOH eye. (**e**) Mean blur rate (MBR) in each quadrant (S, superior; N, nasal; I, inferior; T, temporal) of the optic disc. Red and blue indicate fast and slow blood flow velocities, respectively. The nasal MBR of the optic disc was reduced.

**Figure 2:**
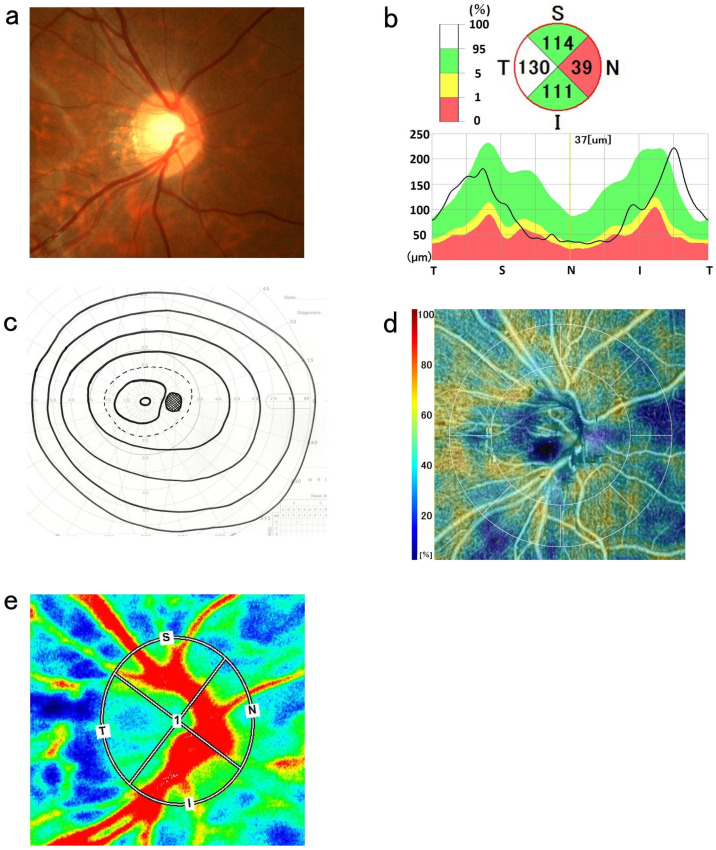
Images showing pseudonasal optic disc hypoplasia in the right eye. (**a**) Fundus photograph showing a double ring-shaped appearance of the nasal optic disc (white arrowheads), a small disc size, and indented nasal disc margin in the nasal peripapillary retina. (**b**) Thickness of the circumpapillary retinal nerve fiber layer (cpRNFL) of the optic disc using optical coherence tomography and thinning of the nasal cpRNFL thickness. (**c**) The Goldman perimetry test showed normal visual fields. (**d**) The color map of radial papillary capillary (RPC) density shows a moderate decrease in RPC density in the nasal region. (**e**) The nasal mean blur rate of the optic disc was mild reduced. Mean blur rate (MBR) in each quadrant (S, superior; N, nasal; I, inferior; T, temporal) of the optic disc. Red and blue indicate fast and slow blood flow velocities, respectively. The nasal MBR of the optic disc was reduced.

An optic disc with a DM/DD ratio > 3.0 was classified as a small optic disc [8]. cpRNFL thickness was divided into four quadrants: superior, temporal, inferior, and nasal. The center of the measurement area was determined using the automatic detection software of RS-3000 Advance 2 (updated; version 1.10.0). For eyes in which it was difficult to detect the center of the optic disc or in which the center was clearly misaligned, an experienced examiner (Y.H.) manually verified and adjusted the center position while referring to the fundus photographs to ensure alignment with the center of the optic disc. cpRNFL thickness was classified on the basis of the standard database: green, within the normal range; yellow, abnormal below the 5% level; and red, abnormal below the 1% level (Figure 1b and Figure 2b) [3,4]. The cpRNFL thickness was corrected using AL. When abnormalities in nasal cpRNFL thickness were observed (indicated by yellow or red in the standard database), a dynamic visual field test using Goldmann perimetry (GP) was also performed.

In this study, the diagnostic criteria for NOH were based on previously reported criteria: (1) a small optic disc, (2) pallor of the nasal optic disc or irregularity of the optic disc margins, (3) wedge-shaped defects temporally extending from Mariotte’s blind spots [7], and (4) a decrease in cpRNFL thickness on the nasal optic disc [3,4] (Figure 1). Cases fulfilling criteria 1, 2, and 4 without visual field abnormalities were defined as pseudo-NOH (pNOH) (Figure 2). In the current study, pNOH was used to classify cases that exhibit anatomical features but do not have visual field abnormalities. All other patients with no abnormalities in the visual field or cpRNFL thickness were classified as normal. Diagnoses were made by an ophthalmologist.

### 2.4. OCTA Measurements

OCTA (RS-3000 Advance 2) was used to quantify RPC density. The Nidek RS-3000 Advance 2 OCT system and AngioScan (updated version 1.8.0) were used to evaluate the OCTA images [4,9]. OCTA scans of the optic disc were conducted with a 4.5 mm × 4.5 mm diameter and a 256 B-scan composition. The RPC density slab thickness for the RS-3000 Advance 2 system encompassed the entire nerve fiber layer from the internal limiting membrane to the nerve fiber layer/ganglion cell layer. The RPC density could be quantified separately and evaluated for up to eight sectors. These eight sectors were quantified as superior, temporal, inferior, and nasal (Figure 1d and Figure 2d).

### 2.5. LSFG Measurements

The LSFG was used to measure the blood flow dynamics at the posterior pole [10,11]. The fundus was illuminated using an 830 nm diode laser to detect moving red blood cells in the retinal vessels [12,13]. LSFG measurements were successively performed three times. The laser speckle method is non-invasive, quantitative, and repeatable [11,14]. It has been reported that this method can be used to measure ocular blood flow velocity and produce reproducible results. Retinal blood flow velocity was evaluated by measuring the optic disc (Figure 1e and Figure 2e) [3,15,16]. When conducting follow-up studies of individuals, each circle was automatically set using LSFG Analyzer version 3.0.47 (Softcare Ltd., Fukuoka, Japan) at the same location where the circle was set at the baseline. The circular area for analyzing the mean blur rate (MBR) was set to the size of the optic disc, and its position was determined using fundus photography. The MBR is the relative value of the blood flow velocity. Each MBR was automatically calculated using the software, and the tissue MBR of the optic disc was analyzed by dividing it into four quadrants (superior, temporal, inferior, and nasal) (Figure 1e and Figure 2e).

### 2.6. Statistical Analyses

All results are expressed as mean ± standard deviation. The Kruskal–Wallis and Steel–Dwass tests were used to examine sequential changes in age, spherical equivalent (SE), AL, IOP, DM/DD ratio, cpRNFL thickness, RPC density, and MBR. To investigate the relationship between RPC density and cpRNFL thickness or MBR, statistical analyses were performed using Spearman’s rank correlation test for each parameter in the four quadrants (superior, temporal, inferior, and nasal). For all tests, *p* < 0.05 indicated statistical significance. A post hoc power analysis was performed using G*Power (version 3.1). Because the primary analysis involved a nonparametric comparison among three independent groups using the Kruskal–Wallis test, the power analysis was conducted based on a one-way ANOVA model as an approximation. The effect size (f = 0.72) was derived from the Kruskal–Wallis H statistic (H = 16) and used to calculate the achieved power. With an α level of 0.05 and a total sample size of 44, the achieved power was >0.99.

## 3. Results

Thirty-six volunteers were enrolled, and the mean age was 22.0 ± 4.3 years. All participants had a BCVA score ≥ 20/20.

### 3.1. Classification Based on Fundus Findings, cpRNFL Thickness, and Visual Field Tests

Fundus findings revealed a small optic disc, irregularity at the margins of the nasal optic disc, and thinning of the nasal cpRNFL in 16 eyes. Among these 20 eyes, seven eyes showed wedge-shaped visual field abnormalities associated with Marriott’s blind spot in Humphrey’s visual field test and/or GP test. Therefore, 7 and 13 eyes were classified as NOH and pNOH, respectively. In this study, 24 right eyes of 24 normal participants matched for age, SE, and AL to the NOH and pNOH groups were used as controls (*p* = 0.599, *p* = 0.173, and *p* = 0.309, respectively) (Table 1). There were no significant differences in age, SE, AL, or IOP among the NOH, pNOH, and normal control groups. The DM/DD ratio was significantly higher in the NOH and pNOH groups than in the normal control group (*p* ≤ 0.001 and *p* < 0.001, respectively); however, the DM/DD ratio did not significantly differ between the NOH and pNOH groups (*p* = 0.221) (Table 1). In addition, inverted disc vessels were observed in four-eyes in the NOH group and in five eyes in the pNOH group. The thickness of the nasal cpRNFL in the NOH and pNOH groups was significantly lower than that in the normal control group (*p* = 0.001 and *p* < 0.001, respectively); however, there was no significant difference in nasal cpRNFL thickness between the NOH and pNOH groups (*p* = 0.241) (Table 1). There were no significant differences between any of the groups in other sectors (Table 1).

### 3.2. RPC Density Data

The nasal RPC density of the NOH eyes was significantly lower than that of the pNOH and normal control groups (*p* = 0.003 and *p* = 0.001, respectively); however, there was no significant difference in nasal RPC density between the pNOH and normal control groups (*p* = 0.065) (Table 1). The superior and inferior RPC densities in the NOH group were significantly lower than those in the control group (*p* = 0.047 and *p* = 0.004, respectively). Furthermore, no significant differences were observed between the groups in the temporal region (Table 1).

### 3.3. MBR Data

The nasal MBR of the NOH and pNOH eyes was significantly lower than that of the control group (*p* = 0.023 and *p* = 0.012, respectively), whereas the nasal MBR did not significantly differ between the NOH and pNOH groups (*p* = 0.929) (Table 1). No significant differences were found between any of the groups in other sectors (Table 1).

### 3.4. Correlation Between RPC Density and cpRNFL Thickness or MBR

In the correlation analysis involving all 44 eyes, there was a significant positive correlation between nasal RPC density and nasal cpRNFL thickness (*R* = 0.700, *p* < 0.001) or nasal MBR (*R* = 0.371, *p* = 0.012) (Table 2). There were no significant correlations among the other factors (Table 2).

## 4. Discussion

In this study, the cpRNFL thickness and MBR of the nasal optic disc were significantly decreased in the NOH and pNOH groups compared with those in the control group; however, there was no significant difference between the NOH and pNOH groups. In addition, the RPC density of the nasal optic disc was significantly lower in the NOH group than in the pNOH and control groups.

The cpRNFL thickness of the nasal optic disc is reduced in eyes with NOH [3,4,5,6]. Ohguro et al. reported that the average nasal cpRNFL thickness in eyes with NOH was 29.8 µm and was markedly reduced in eyes with unilateral NOH (19.5 µm) compared with that with glaucoma, which causes visual field abnormalities on the temporal side [5]. Haruta et al. also showed that small or tilted optic discs were associated with NOH and that the average nasal cpRNFL thickness was reduced (34.8 µm) [6]. In addition, Hasegawa et al. reported that the ratio of nasal cpRNFL thickness to nasal MBR in the eyes of patients with NOH was significantly reduced compared with that of normal control eyes; they also stated that there was a significant correlation between nasal cpRNFL thickness and MBR [3]. In a recent study, OCTA was performed on an eye with NOH and revealed markedly reduced nasal RPC density. Interestingly, this report showed that the fellow eye, in which NOH was suspected because of fundus findings and nasal cpRNFL thinning, had no visual field abnormalities, and the decrease in nasal RPC density was less than that in the NOH eye [4].

There are various reports on superior segmental optic disc hypoplasia (SSOH), which is the most common type of optic disc hypoplasia [17,18,19]. Unoki et al. showed that the mean cpRNFL thickness of the superior quadrant in normal control eyes was 127.3 μm, whereas it was markedly reduced at 56.7 μm in SSOH eyes [18]. Aizawa et al. used LSFG and reported that patients with SSOH had significantly reduced superior cpRNFL thickness compared with those with normal eyes and that the blood flow velocity in the optic disc was reduced in all quadrants, including the superior quadrant [19]. Furthermore, patients with SSOH have lower RPC density than normal participants in three quadrants, except for the temporal quadrant, and the diagnostic power of RPC density was the highest in the superior quadrant [2].

In this study, there were no significant differences in nasal cpRNFL thickness or nasal optic disc MBR between NOH and pNOH eyes. Meanwhile, nasal RPC density was significantly lower in the NOH group than in the pNOH group. However, there was no significant difference in nasal RPC density between the pNOH and control groups. Therefore, these results revealed that even when NOH was suspected on the basis of the fundus findings, OCT C-scan, and LSFG images, differences existed in the presence of visual field abnormalities. The density of RPC may be responsible for these differences. Additionally, the inferior RPC density in the NOH group was significantly lower than that in the normal group, thus suggesting a congenital decrease in blood vessel density in the inferior region of the NOH eye.

This study has a few limitations. First, although the statistical power was higher than the desired threshold (99.9% vs. 80.0%), the sample size in the NOH group was actually small, thus leading to an extremely preliminary assessment based on individual cases; consequently, the results of the three-group comparisons and interpretation of correlations are limited, and a prospective cross-sectional study design is required. Second, the underlying pathophysiological mechanism linking reduced RPC density to functional visual field defects in NOH remains unclear. In addition, owing to the narrow age range of the participants in this study, it was difficult to generalize these findings to older clinical populations. Given that retinal morphology and circulatory dynamics may be influenced by aging [20,21,22], further studies involving a diverse range of age groups are necessary to verify the utility of cpRNFL thickness, RPC density, and MBR as clinical biomarkers for assessing NOH in ophthalmic practice. In the future, the number of NOH eyes should be increased, and large-scale studies should be conducted. Longitudinal studies are also necessary to observe whether there are any changes in the findings or examination results or whether glaucoma develops. In addition, inverted disc vessels were observed in both the NOH and pNOH groups. However, considering that no previous reports have described inverted disc vessels as diagnostic criteria or characteristic findings for NOH, it is unclear whether they influence the measurement results. Nevertheless, they may be worthy of attention in future studies. We hope that the underlying pathophysiological mechanisms linking reduced RPC density to functional visual field defects in NOH will be elucidated.

## 5. Conclusions

In conclusion, even if examination findings similar to those of NOH are observed, such as small optic disc, optic disc margin irregularity, reduced nasal cpRNFL thickness on OCT, or reduced nasal optic disc blood flow velocity on LSFG, nasal RPC density may be involved in the presence or absence of visual field abnormalities. This finding suggests that pNOH and NOH may indicate differences in disease severity. It may be possible to detect NOH and assess its severity by evaluating RPC density via OCTA; however, further detailed investigations are needed.

## Figures and Tables

**Table 1:** Comparison of age and ophthalmologic data in participants with NOH, pNOH, and normal controls.

	NOH Group	pNOH Group	Normal Group	Kruskal–Wallis Test	Steel–Dwass Test (*p* Value)		
				(*p* Value)	NOH vs. pNOH	NOH vs. Normal	pNOH vs. Normal
Age (years)	21.0 ± 1.5	21.2 ± 0.9	22.2 ± 5.2	0.599	0.780	0.996	0.573
SE (D)	−4.02 ± 2.52	−3.83 ± 1.97	−2.66 ± 2.49	0.173	0.997	0.409	0.207
AL (mm)	25.61 ± 1.20	25.52 ± 1.19	25.06 ± 1.02	0.309	0.929	0.411	0.455
IOP (mmHg)	14.6 ± 2.0	14.4 ± 3.0	14.0 ± 2.4	0.864	0.838	0.901	0.967
DM/DD ratio	3.42 ± 0.31	3.20 ± 0.12	2.63 ± 0.25	<0.001	0.221	<0.001	<0.001
cpRNFL thickness (µm)							
Superior	124.6 ± 9.4	136.9 ± 11.7	130.2 ± 12.2	0.113	0.092	0.585	0.321
Temporal	105.6 ±16.2	95.3 ± 14.6	90.0 ± 18.6	0.060	0.357	0.083	0.328
Inferior	127.0 ± 12.7	129.8 ± 14.8	130.1 ± 13.6	0.693	0.956	0.570	0.967
Nasal	41.9 ± 5.9	47.2 ± 5.7	65.1 ± 13.3	<0.001	0.241	0.001	<0.001
RPC density (%)							
Superior	50.9 ± 5.7	53.8 ± 2.4	56.5 ± 3.3	0.012	0.647	0.047	0.051
Temporal	52.4 ± 7.8	55.5 ± 3.4	54.6 ± 5.1	0.930	0.929	0.961	0.975
Inferior	44.6 ± 7.1	52.4 ± 5.7	55.0 ± 5.4	0.002	0.063	0.004	0.157
Nasal	19.2 ± 5.6	39.0 ± 10.0	45.8 ± 11.3	<0.001	0.003	0.001	0.065
MBR							
Superior	13.2 ± 1.4	13.7 ± 2.2	14.7 ± 2.2	0.088	0.977	0.196	0.1577
Temporal	10.1 ± 2.1	11.2 ± 2.5	11.4 ± 1.8	0.528	0.838	0.481	0.862
Inferior	13.2 ± 1.3	13.9 ± 2.2	14.6 ± 1.9	0.147	0.968	0.205	0.298
Nasal	11.6 ± 1.5	12.1 ± 2.0	13.8 ± 1.8	0.003	0.929	0.023	0.012

Mean ± SD. NOH, nasal optic disc hypoplasia; pNOH, pseudonasal optic disc hypoplasia; SE, spherical equivalent; AL, axial length; IOP, intraocular pressure; DM/DD, disc–macula distance to disc diameter; cpRNFL, circumpapillary retinal nerve fiber layer; RPC, radial peripapillary capillaries; MBR, mean blur rate; SD, standard deviation. Kruskal–Wallis test, Steel–Dwass test.

**Table 2:** Correlation among RPC density, cpRNFLT, and MBR in all 44 eyes.

Superior	RPC Density		Temporal	RPC Density		Inferior	RPC Density		Nasal	RPC Density	
	Coefficient	*p* Value		Coefficient	*p* Value		Coefficient	*p* Value		Coefficient	*p* Value
cpRFNLT	0.040	0.793	cpRFNLT	0.139	0.367	cpRFNLT	0.100	0.514	cpRFNLT	0.700	<0.001
MBR	0.215	0.159	MBR	0.027	0.858	MBR	0.261	0.086	MBR	0.371	0.012

RPC, radial peripapillary capillary; cpRNFLT, circumpapillary retinal nerve fiber layer thickness; MBR, mean blur rate. Spearman’s rank correlation test.

## Data Availability

The data presented in this study are available on request from the corresponding author (the data are not publicly available due to privacy or ethical restrictions).

## Abbreviations

The following abbreviations are used in this manuscript:

AL	Axial length
BCVA	Best-corrected visual acuity
cpRNFL	Circumpapillary retinal nerve fiber layer
DM/DD	Disc–macula distance to disc diameter
GP	Goldmann perimetry
IOP	Intraocular pressure
LSFG	Laser speckle flowgraphy
MBR	Mean blur rate
NOH	Nasal optic disc hypoplasia
OCT	Optical coherence tomography
OCTA	Optical coherence tomography angiography
pNOH	Pseudonasal optic disc hypoplasia
RPC	Radial peripapillary capillary
SE	Spherical equivalent
